# *Anopheles* bionomics in a malaria endemic area of southern Thailand

**DOI:** 10.1186/s13071-021-04870-8

**Published:** 2021-07-27

**Authors:** Narenrit Wamaket, Oranicha Khamprapa, Sittinont Chainarin, Panisa Thamsawet, Ubolrat Ninsaeng, Suttipong Thongsalee, Veerast Suwan, Jira Sakolvaree, Ratree Takhampunya, Silas A. Davidson, Patrick W. McCardle, Patiwat Sa-angchai, Mavuto Mukaka, Kirakorn Kiattibutr, Amnat Khamsiriwatchara, Wang Nguitragool, Jetsumon Sattabongkot, Jeeraphat Sirichaisinthop, Kevin C. Kobylinski

**Affiliations:** 1 Department of Entomology, Armed Forces Research Institute of Medical Sciences, Ratchathewi, Bangkok, Thailand; 2 Mahidol Vivax Research Unit, Faculty of Tropical Medicine, Mahidol University, Ratchathewi, Bangkok, Thailand; 3 Surat Thani Vector-Borne Diseases Control Center 11.3, Muang, Surat Thani, Thailand; 4 Department of Tropical Hygiene, Faculty of Tropical Medicine, Mahidol University, Ratchathewi, Bangkok, Thailand; 5 Mahidol-Oxford Tropical Medicine Research Unit, Faculty of Tropical Medicine, Mahidol University, Ratchathewi, Bangkok, Thailand; 6 Centre for Tropical Medicine and Global Health, Nuffield Department of Clinical Medicine, University of Oxford, Oxford, UK; 7 Center of Excellence for Biomedical and Public Health Informatics, Faculty of Tropical Medicine, Mahidol University, Ratchathewi, Bangkok, Thailand; 8 Department of Molecular Tropical Medicine and Genetics, Faculty of Tropical Medicine, Mahidol University, Ratchathewi, Bangkok, Thailand; 9 Department of Disease Control, Ministry of Public Health, Muang, Nonthaburi, Thailand

**Keywords:** *Anopheles*, *Plasmodium*, Malaria, Thailand, Surat Thani, Parity

## Abstract

**Background:**

Ivermectin mass drug administration (MDA) could accelerate malaria elimination in the Greater Mekong Subregion. This study was performed to characterize the bionomics of *Anopheles* in Surat Thani province, Thailand.

**Methods:**

Mosquitoes were collected via human landing collections between February and October 2019. *Anopheles* mosquitoes were morphologically identified to species. Primary *Anopheles* malaria vectors were dissected to assess parity status, and a subset were evaluated for molecular identification and *Plasmodium* detection.

**Results:**

A total of 17,348 mosquitoes were collected during the study period; of these, 5777 were *Anopheles* mosquitoes. Morphological studies identified 15 *Anopheles* species, of which the most abundant were *Anopheles minimus *(s.l.) (87.16%, *n* = 5035), *An. dirus* s.l. (7.05%, *n* = 407) and *An. barbirostris* s.l. (2.86%, *n* = 165). Molecular identification confirmed that of the *An. minimus* s.l. mosquitoes collected, 99.80% were *An. minimus* (s.s.) (*n* = 484) and 0.2% were *An. aconitus* (*n* = 1), of the *An. dirus* (s.l.) collected, 100% were *An. baimaii* (*n* = 348), and of the *An. maculatus* (s.l.) collected, 93.62% were *An. maculatus* (s.s.) (*n* = 44) and 6.38% were *An. sawadwongporni* (*n* = 3). No *Anopheles* mosquito tested was *Plasmodium* positive (0/879). An average of 11.46 *Anopheles* were captured per collector per night. There were differences between species in hour of collection (Kruskal–Wallis H-test:* χ*^2^ =  80.89, *P* < 0.0001, *n* = 5666), with more *An. barbirostris* (s.l.) and *An. maculatus* (s.l.) caught earlier compared to *An. minimus* (s.l.) (*P* = 0.0001 and *P* < 0.0001, respectively) and *An. dirus* (s.l.) (*P* = 0.0082 and *P* < 0.001, respectively). The proportion of parous *An. minimus* (s.l.) captured by hour increased throughout the night (Wald Chi-square: *χ*^2^ = 17.31, *P* = 0.000, odds ratio = 1.0535, 95% confidence interval 1.0279–1.0796, *n* = 3400). Overall, *An. minimus* (s.l.) parity was 67.68% (2375/3509) with an intra-cluster correlation of 0.0378. A power calculation determined that an *An. minimus* (s.l.) parity reduction treatment effect size = 34%, with four clusters per treatment arm and a minimum of 300 mosquitoes dissected per cluster, at an *α* = 0.05, will provide 82% power to detect a significant difference following ivermectin MDA.

**Conclusions:**

The study area in Surat Thani province is an ideal location to evaluate the impact of ivermectin MDA on *An. minimus* parity.

**Graphical abstract:**

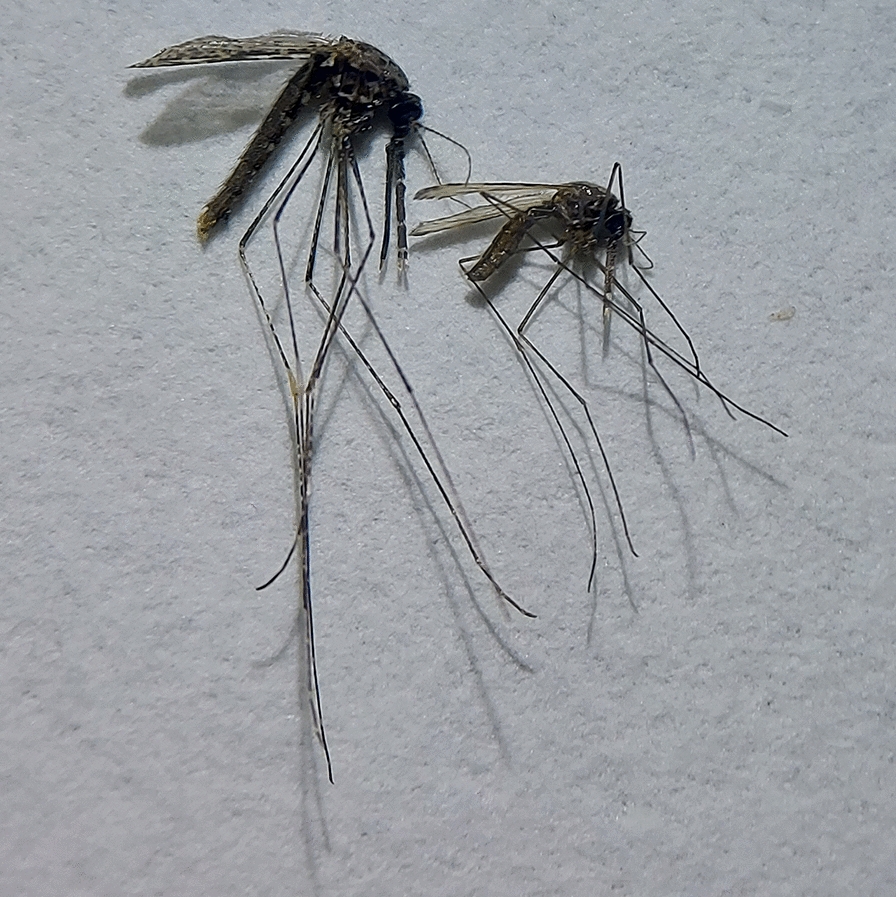

## Background

In 2019, an estimated 229 million cases of malaria occurred worldwide, with approximately 239,000 cases reported from the Greater Mekong Subregion (GMS). Between 2010 and 2019, the number of malaria cases in the GMS fell by 90%, and the target of the countries within the GMS is to eliminate malaria by 2030 [1]. Malaria transmission in the GMS is complex, with various *Anopheles* species in the Dirus complex, Minimus complex and the Maculatus group having been recognized as the primary malaria vectors in the region. From the Minimus complex, *Anopheles minimus* (s.s.) is the primary malaria vector and can be found across Thailand and the GMS [2–6]. There are two members of the *An. dirus* complex that are primary malaria vectors; of these, *An. dirus* (s.s.) occurs east of the Thai–Myanmar border, and *An. baimaii* occurs west of the Thai–Myanmar border [2, 6, 7]. Additionally, members of the *An. maculatus* group are considered to be primary vectors that contribute to malaria transmission, including *An. maculatus* (s.s.) [2, 4, 5] and to a lesser extent *An. sawadwongporni* [2, 4, 6], with the latter identified as a malaria vector in southern Thailand [2]. Members within a species complex differ in their behavioral characteristics, which in turn drives *Plasmodium* transmission dynamics; therefore, an accurate identification of mosquitoes is essential to design and evaluate vector control methods in the GMS.

The diversity of vector species, insecticide resistance and increasing antimalarial drug resistance are some of the greatest challenges for malaria elimination in the GMS [8]. Moreover, the outdoor-feeding, outdoor-resting and early-evening feeding behaviors of GMS malaria vectors [9–11] also limit the effectiveness of indoor residual spraying (IRS) and long-lasting insecticide-treated nets (LLINs). Therefore, novel vector control measures which target these outdoor-feeding vectors could accelerate malaria elimination in the GMS. The mass drug administration (MDA) of ivermectin to humans has been suggested as a possible malaria parasite transmission control tool as it directly kills *Anopheles* that feed on treated people, regardless of blood-feeding time or location. Evidence from West Africa has shown that a single ivermectin MDA can kill wild *An. gambiae* (s.l.) [12, 13], shift the population age structure [13], reduce the *Plasmodium falciparum* sporozoite rate [13, 14] and that repeated ivermectin MDAs reduce clinical falciparum malaria episodes [15]. In the GMS, at human-relevant concentrations, ivermectin is lethal to *An. dirus*,* An. minimus*, *An. sawadwongporni* and *An. campestris*, and it inhibits the development (*i.e.* sporogony) of *Plasmodium vivax* in *An. dirus* and *An. minimus* [16]. A clinical trial demonstrated that *An. dirus* fed blood from persons treated with ivermectin (400 µg/kg) within 6 days post ivermectin administration had a 50% reduced survival rate; the reduction in *An. minimus* survival increased to 90% when they were fed blood from treated persons up to 10 days post drug administration [17]. This evidence has inspired a large-scale cluster randomized trial in Thailand to assess the effect of ivermectin MDA on entomological and parasitological parameters of malaria transmission.

Evaluating vector control interventions for malaria in the GMS is difficult as transmission occurs primarily in the forest due to *Anopheles* habitat preference, combined with sporadic human entry into the forest for various agricultural and economic pursuits, not all of which are legal [18, 19]. However, most vector control interventions (*e.g.* LLINs and IRS) are applied in the village setting where transmission is less likely to occur, and thus they do not directly target the areas of active *Plasmodium* transmission. Thailand has dramatically reduced its malaria burden and has set the goal to eliminate malaria by 2024. Indeed, from 2000 to 2019, there was a 96% reduction in number of malaria cases, from 159,120 to 5832, but the ratio of Thai to non-Thai cases has increased from 57 to 72% demonstrating that there are still active foci of transmission in Thailand [20]. This reduction in malaria complicates evaluation of *Anopheles* vector control interventions in Thailand due to low rates of *Plasmodium* transmission. To evaluate the effect of ivermectin MDA, a site in Thailand needed to be selected where persons live and work with active malaria transmission. Rubber plantations in Thailand offer an ideal location for *Anopheles* vector control intervention evaluation, as mature rubber plantations tend to be located in hilly areas adjacent to natural forests, both of which are ideal habitats for primary malaria vector proliferation. Rubber tappers live and work in the same environment, and tappers work throughout the night exposed to wild *Anopheles*; consequently, rubber tappers have higher rates of malaria than non-rubber tappers [21–23]. Indeed, a seven-province wide survey of case data from malaria clinics in Thailand from 2013 to 2016 found that 60.1% (3330/5541) of *P. falciparum* cases were identified from rubber tappers [23].

Of all the provinces in Thailand, Surat Thani province has the largest rubber plantation coverage, approximately 3829 km^2^ [24], and is one of the few provinces affected by malaria that is not along an international border. Interestingly, in Surat Thani, the dominant malaria species infecting humans is *P. falciparum*, accounting for 77.58% (519/669) of all malaria cases from 2015 to 2019 [25]. *Plasmodium falciparum* is the ideal parasite to assess during vector control interventions as it is most sensitive to transmission interruption due to its non-relapsing nature. A cross-sectional molecular malaria survey conducted in Surat Thani in 2019 demonstrated that persons who stayed outdoors during the night-time were at the highest risk of malaria infection [26]. In that study, three districts in Surat Thani province, namely the Phanom, Vibhavadi and Khiri Rat Nikhom districts, were selected for evaluation based on their malaria case incidence comprising 65.13% (338/519) *P. falciparum* cases between 2015 and 2019 and high rubber plantation coverage. Historically, IRS with 5% deltamethrin has been performed in villages with a higher malaria case burden, but this control measure ceased in Phanom and Vibhavadi in 2015 and in Khiri Rat Nikhom in 2016. LLINs are widely distributed throughout all three districts by government and non-governmental organizations. Daytime indoor thermal fogging for *Aedes aegypti* control with 1% deltamethrin or 25% cypermethrin still occurs focally and sporadically in response to local dengue cases (personal communication, Surat Thani Vector-borne Diseases Control Center 11.3, Surat Thani, Thailand).

Little information regarding *Anopheles* vector bionomics in Surat Thani has been published. The largest study conducted in Surat Thani to date reported a total of 3778 *Anopheles* mosquitoes collected from February 2015 to December 2016 via human landing collection (HLC) in Phanom district [27]. In the same study, six *Anopheles* species were collected, including *An. dirus* (s.l.), *An. minimus* (s.l.), *An. maculatus* (s.l.), *An. barbirostris* (s.l.), *An. hycranus* (s.l.) and *An. tessellatus*. The predominant species was *An. minimus *(s.l.), comprising 87.19% of *Anopheles* collected. The highest mosquito densities were found between March and May in both years [27]. Molecular identification has verified the presence of *An. maculatus* (s.s.) in Phanom district [28] and *An. minimus* (s.s.) in Khiri Rat Nikhom district [29]. Due to active *P. falciparum* transmission, asymptomatic malaria observation, presence of primary malaria vectors and an ideal environment for evaluating a vector control intervention, Surat Thani province was selected as the study area to evaluate the impact of ivermectin MDA on entomological and parasitological parameters. Due to decreasing rates of *Plasmodium* transmission in Thailand, entomological (i.e. mosquito population age structure) and parasitological (i.e. human malaria prevalence) outcomes will be used to assess ivermectin MDAs. The unexpectedly low prevalence of malaria in the study sites [26] necessitates a strong emphasis on the former.

Baseline entomological surveillance utilizing the mosquito HLC method were performed to evaluate vector abundance, composition, landing activity and parity rates linked to molecular identification in Surat Thani in 2019. These efforts were undertaken to determine whether the study area was appropriate to evaluate the impact of ivermectin MDA on *An. minimus* population age structure (i.e. parity).

## Methods

### Ethics statement

This study was approved by the Walter Reed Army Institute of Research (WRAIR #2430), the Human Research Protection Office (HRPO Log No. 19919.2a/A-19919.2b) and the Ethical Review Committee for Research in Human Subjects, Ministry of Public Health, Thailand (Thai MoPH Ref No. 25/2560).

### Description and maps of field sites

#### Field site description

Collection sites were located in Khiri Rat Nikhom, Phanom and Vibhavadi districts, in Surat Thani province (651 km south of Bangkok). Villages with higher malaria case burden, as reported by local Vector Borne Disease Units, were selected from each district. Populations and house locations were mapped (see section Mapping process), and cluster sizes of approximately 300–500 persons were established. In total, 13 clusters were selected for entomological evaluation: five clusters in Khiri Rat Nikhom, four clusters in Phanom and four clusters in Vibhavadi. Khiri Rat Nikhom district is located in the center of Surat Thani province (9°1′48″N, 98°57′12″E), with its western part located in the forested hills of the Phuket mountain range adjacent to Khao Sok National Park and its eastern part mostly consisting of flat terrain. Phanom is located in the southwest of the province (8°51′18″N, 98°48′48″E) and is covered by mountains and forest. Its northwestern part is protected by the Khao Sok National Park and its southwestern part is protected by the Khlong Phanom National Park. Vibhavadi is a small district situated in the north-central portion of the province (9°14′20″N, 98°58′44″E) and is covered by mountain and forest. The western part of the district is protected by the Kaeng Krung National Park and Khlong Yan Wildlife Sanctuary. In Surat Thani, the dry season occurs from January to February, and the rainy season lasts from March to September; heavy monsoon rains occur October to December.

#### Mapping process

Latitude and longitude coordinates of all houses and HLC locations were captured using a 60CSx GPS unit (Garmin, Olathe, KS, USA). The open-source QGIS software was used to generate maps for each cluster. The terrain data were derived from a topographic map with elevation contour lines at 25 m.

### Collection, morphological identification and parity evaluation of mosquitoes

#### Mosquito collection

Adult mosquitoes were collected from February to October 2019 for two consecutive nights per cluster per month, with the exception of Vibhavadi where collections began in June. Mosquitoes were collected by the HLC method. Mosquito collector volunteers were local Thai residents, non-pregnant and non-breastfeeding adults (age 18–62 years) who were capable of providing informed consent and capable of comprehending the HLC method. A canopy was constructed at each outdoor collection site to protect the mosquito collector volunteers from the elements. There were two collection sites per cluster each night. Collection sites were chosen based on proximity to a forest or rubber plantation, presence of a house nearby for access to water and electricity and close proximity to potential *An. minimus* (s.l.) larval habitat. Efforts were made to sample as many areas in each cluster that met the above criteria and were safely accessible. If a collection site yielded few or no *Anopheles*, then the site was switched to a new location the following night. At each collection site, two mosquito collector volunteers worked together from 18:00 h to 24:00 h and then replaced by two other collectors from 24:00 h to 06:00 h. Collections occurred for 50 min of each hour, followed by a 10-min break. Volunteers were instructed to wear double-layer clothing. Each collector exposed only their legs and captured mosquitoes as soon as they landed on them using a plastic collection tube sealed with a cotton ball. The mosquitoes were then transferred to collection cups grouped by hour of collection and separated by each volunteer. The cups were kept in a Styrofoam box and covered with moist towel to keep the mosquitoes humid and alive during transport back to the field station.

#### Mosquito morphological identification and parity dissections

At the field station, mosquitoes were transferred to plastic knockdown chambers and anesthetized with triethylamine (Flynap®; Carolina Biological Supply Co., Burlington, NC, USA) for 2 min, following which they were identified morphologically under a dissecting stereomicroscope (Stemi 305; Carl Zeiss AG, Oberkochen, Germany) using a standard key of adult *Anopheles* of Thailand [30]. All primary *Anopheles* species, such as the *An. dirus* complex and *An. maculatus* group, and a subset of 20 *An. minimus* complex per collector pair per site from 18:00 h to 24:00 h and from 24:00 h to 06:00 h were dissected to remove their ovaries. Once mosquitoes were anesthetized, they were placed in plastic Petri dishes and care was taken to keep the mosquitoes in an ice-chilled cooler with a damp towel to maintain humidity. Thus, almost all mosquitoes were alive at the point of ovary dissection. Ovaries were dissected with minuten pins in a drop of bottled Crystal water, a brand of marketed water commonly found throughout Thailand. The last two abdominal segments were gently pulled apart from the abdomen, and the ovaries were separated from the remaining internal organs and transferred to an individual well on a 12-well slide. The slide was then allowed to air dry, and care was taken to observe each ovary before total evaporation of water to assess parity status. Determination of parity status was based on the presence of coiled (nulliparous) and uncoiled (parous) tracheole skeins viewed at ×10 and ×40 magnification with a compound microscope (model B-190TB; Optika Srl, Ponteranica, Italy) and images of the ovaries were taken for reference. On occasion, if the primary reviewer had doubts on the parity status, then a second reviewer was consulted. The mosquito sample was then bisected between the thorax and the abdomen and stored in labeled 2-ml centrifuge tubes with silica gel desiccant. Processed mosquitoes were shipped back to the Armed Forces Research Institute of Medical Sciences in Bangkok for molecular species identification and *Plasmodium* infection evaluation of the thorax.

### Molecular methods for *Anopheles* and *Plasmodium* identification

#### DNA extraction method

The DNA extraction method involved adding 700 µl of phosphate buffer saline (pH 7.4) and 4.5-mm steel beads (Copperhead; Crosman Corporation, Bloomfield, NY, USA) to a 2-ml tube containing an individual mosquito thorax or abdomen, followed by homogenization in the TissueLyser II (Qiagen, Hilden, Germany) at 22 Hz for 2 min. The mosquito suspension was then centrifuged at 12,000 rpm for 5 min, and a 250-µl aliquot of the supernatant was used for DNA extraction according to the QIAsymphony® DNA Minikit and Tissue LC 200 DSP protocol in the fully automated QIAsymphony® SP system (Qiagen). The DNA was eluted in 50 μl and stored at − 20 °C until further use. DNA/RNA-free distilled water was included in the extraction process as a negative extraction control.

#### Molecular methods for *Anopheles* sibling species identification

To identify anopheline sibling species, multiplex allele-specific PCR (AS-PCR) assays were used to examine the internal transcribed spacer 2 (ITS2) region of DNA and distinguish the members of the Dirus complex [*An. dirus* (s.s.), *An. scanloni*, *An. cracens*, *An. baimaii*, *An. nemophilous*] [31], the Maculatus group [*An. maculatus* (s.s.), *An. pseudowillmori*, *An. sawadwongporni*, *An. rampae*, *An. dravidicus*] [32] and the Funestus group [*An. minimus* (s.s.), *An. harrisoni*, *An. aconitus*, *An. varuna*, *An. pampanai*] [33]. Previously published protocols [31–34] were used, with the following modifications. The amplification was carried out using total volumes of 25 μl, with the final optimized reaction conditions as follows: (i) 1× GoldStar Best Master mix (GoldStar DNA Polymerase, dNTP, PCR stabilizer and enhancer); (ii) three specific primer cocktails, each containing four or five different primer pairs to discriminate between species with 400 nM for the primers specific for the Funestus group and Maculatus group and 500 nM for the primers specific for the Dirus complex; (iii) 4% dimethyl sulfoxide (DMSO) was included only for Dirus complex reactions; (iv) a universal forward primer, located in the conserved region of the* 5.8S* gene, and species-specific reverse primers in the ITS2 spacer region were employed to amplify a portion of the mosquito ITS2 region; and (v) 1 μl of genomic DNA was used as template. Positive mosquito controls [i.e.* An. minimus* (s.s.), *An. dirus* (s.s.), *An. sawadwongporni*]) were obtained from mosquito colonies maintained at the Armed Forces Research Institute of Medical Science Department of Entomology. Negative DNA/RNA-free distilled water controls were included.

Amplifications were performed in a T100 DNA thermal cycler (Bio-Rad, Hercules, CA, USA) under the following PCR conditions. For the Maculatus group, the cycling program started with an initial denaturation at 95 °C, 10 min; then denaturation at 94 °C/1 min, primer annealing at 55 °C/30 s, extension at 72 °C/30 s for 35 cycles; and a final extension at 72 °C for 10 min. The amplification conditions for the Dirus complex were the same as those for the Maculatus group, except that the annealing time was 15 s instead of 30 s. For the Funestus group, which contains the Minimus complex, the cycling program started with an initial denaturation at 95 °C, 10 min; then amplification at 94 °C/30 s, 45 °C/30 s, 72 °C/40 s for 35 cycles; and a final extension at 72 °C for 10 min. The amplified PCR products were subjected to DNA fragment analysis using the QIAxcel Advanced System (Qiagen) according to the manufacturer’s instructions. Briefly, 10 μl of PCR product was analyzed with the QIAxcel DNA Fast Analysis Cartridge (Qiagen), using the DM190 method and QX 15-bp/1-kb alignment markers. Fragment sizes were calculated using the BioCalculator (Qiagen).

#### DNA sequencing and data analysis

To confirm the results of multiplex AS-PCR assays, a representative of each anopheline group was selected for confirmation with the ITS2 rDNA gene in the DNA sequencing assays. Amplification of the ITS2 rDNA gene from mosquito DNA extracts was conducted using universal primer ITS2A (5′-TGT GAA CTG CAG GAC ACA T-3′) and ITS2B (5′-TAT GCT TAA ATT CAG GGG GT-3′) [35, 36]. Reactions were performed in a T100 DNA thermal cycler (Bio-Rad). PCR reaction mixtures (25 μl) consisted of 2 µl of mosquito DNA extract, 0.1 U of AmpliTaq® Gold DNA Polymerase (Life Technologies, Thermo Fisher Scientific, Carlsbad, CA, USA), 1× Gold buffer, 0.2 mM of dNTP, 2 mM of MgCl_2_ and 0.2 µM of each primer. The PCR cycling program consisted of an initial denaturation step at 94 °C, 10 min; then 94 °C/30 s, 60 °C/1 min, 72 °C/1 min for 37 cycles; and a final extension step at 72 °C for 5 min. The size of the PCR product was determined using the QIAxcel Advanced System as described above. The PCR product was cleaned by the ExoSAP-IT™ PCR Products Clean-up kit: 2 µl of ExoSAP-IT™ was added directly to 5 µl of PCR reaction product, incubated at 37 °C for 15 min and then at 80 °C for 15 min. The ITS2 rDNA gene PCR product was sequenced using the Bigdye® Terminator v3.1 Cycle Sequencing Kit (Applied Biosystems, Waltham, MA, USA) according to Applied Biosystems’ protocol with forward and reverse universal ITS2A and ITS2B primers and run on a SeqStudio Genetic Analyzer (Life Technologies). Forward and reverse nucleotide sequence data for each sample were assembled using the Sequencher 5.1 software package (Gene Code Corp., Ann Arbor, MI, USA). The *Anopheles* species were identified by phylogenetic analysis. Briefly, the MUSCLE algorithm was used for sequence alignment in Molecular Evolutionary Genetics Analysis (MEGA) 6.0 software [37]. Maximum likelihood trees were constructed with the best fit model of nucleotide substitution with bootstrapping (1000 replicates) using MEGA 6.0 software as described by Tamura et al. [37].

#### Real-time PCR for detection of Plasmodium

Real-time PCR for *Plasmodium* detection from *Anopheles* thoraxes was performed using a 7500 Fast Real-time PCR System (Life Technologies). The primers and probes were modified from a previously published protocol of Kimura et al. [38] using the* 18S* subunit of rRNA of *Plasmodium* spp. as a target gene as follows: forward P2F (5′-TAT TCA GAT GTC AGA GGT GAA ATT C-3′), reverse P2R (5′-GAA CCC AAA GAC TTT GAT TTC TCA T-3′) and *Plasmodium* Genus Probe (5′-FAM- ACG ATC AGA TAC CGT CGT AAT CTT-BHQ2-3′). The real-time PCR reaction (25 µl) consisted of 10 µl of KAPA PROBE FAST qPCR Master Mix (2×) (Roche, Branford, CT, USA) containing KAPA Taq HotStart DNA Polymerase, dNTPs, MgCl_2_, stabilizers, 0.3 µl of each 20 µM primer, 1 µl of 10 µM probe, 1 µl of mosquito DNA extract and 8.2 µl of nuclease-free water. *Plasmodium falciparum*- and *P. vivax*-infected *An. dirus* (s.s.) served as positive controls and uninfected *An. dirus* as negative controls and were included in every run. The thermocycler conditions included initial steps at 50 °C, 2 min and 95 °C, 2 min; then amplification at 95 °C/15 min, 60 °C/30 min for 40 cycles; the cut-off values were set automatically at every run. All positive samples were confirmed for *Plasmodium* species by nested PCR.

#### Nested PCR Analysis for *Plasmodium* species differentiation

Nested PCR was performed using two amplification processes as described by Kimura et al. [38] to identify four *Plasmodium *species: *P. falciparum*, *P. vivax*, *P. ovale* and *P. malariae*. The nested PCR was performed in a T100 DNA thermal cycler (Bio-Rad). For the first PCR reaction, the reaction mixture (20 µl) consisted of 1× PCR Gold Buffer II (50 mM KCl, 15 mM Tris–HCl, pH 8.0), 1.5 mM MgCl_2_, 200 μM dNTP, 0.4 μM of each specific outer primer set (P1F and P2R), 0.25 U of Amplitaq Gold™ DNA polymerase and 1 µl of DNA template. The cycling conditions of the first PCR were: 94 °C, 10 min; then 92 °C/30 s, 60 °C/90 s, 72 °C/1 min for 35 cycles. Nuclease-free water was used as a negative control. The amplified fragment was analyzed using the Qiaxcel Advanced System with an expected size of around 140–160 bp. For the second PCR, the product of the first PCR reaction was diluted (1:50) with nuclease-free water and used as a template in the second PCR reaction. The reaction mixture of the second PCR reaction (20 µl) and the cycling program were the same as those of the first PCR reaction, except that the reverse specific primers were used instead of the P2R primer. The expected product (about 110 bp) of each *Plasmodium* species was determined using the Qiaxel Advanced System as described above.

### Statistical analysis

Mosquito diversity per cluster was estimated using the Shannon–Wiener index [39] and the Simpson index [40]. The number of *Anopheles* caught per night was calculated by assessing the total number of *Anopheles* captured at one HLC site divided by two for the pair of collectors used at each location; this number was then used to calculate the mean number of *Anopheles*. The median mosquito catching time was calculated, and the comparison of the distribution of mosquito catching times among groups were assessed using the non-parametric Kruskal–Wallis H-test; pairwise comparisons for significantly different groupings were performed using the post-hoc Dunn’s multiple comparisons test to account for the multiple comparisons using Prism version 7.2 (GraphPad Software, San Diego, CA, USA). Parity by cluster and hour was assessed by a logistic regression model, and the clustering of outcomes was accounted for by using the robust standard errors. The sample size and power calculations for a cluster randomized trial were performed using the clustersampsi command in STATA version 14 (StataCorp, College Station, TX, USA).

## Results

### Topographical maps

**Fig. 1:**
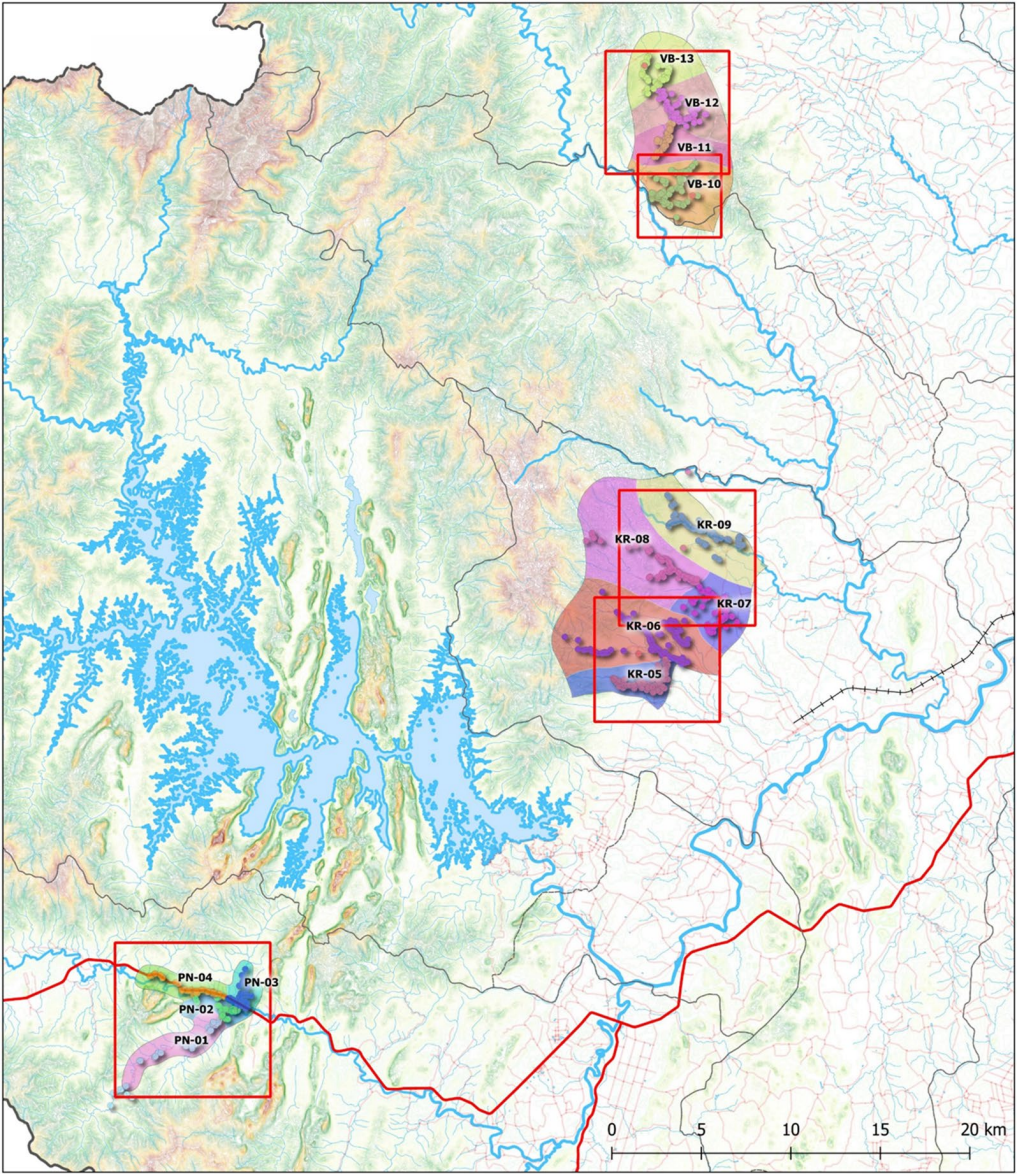
Cluster locations that were surveyed for *Anopheles* mosquitoes in 2019 and surrounding terrain features.* KR* Khiri Rat Nikhom,* PN* Phanom,* VB* Vibhavadi

**Fig. 2:**
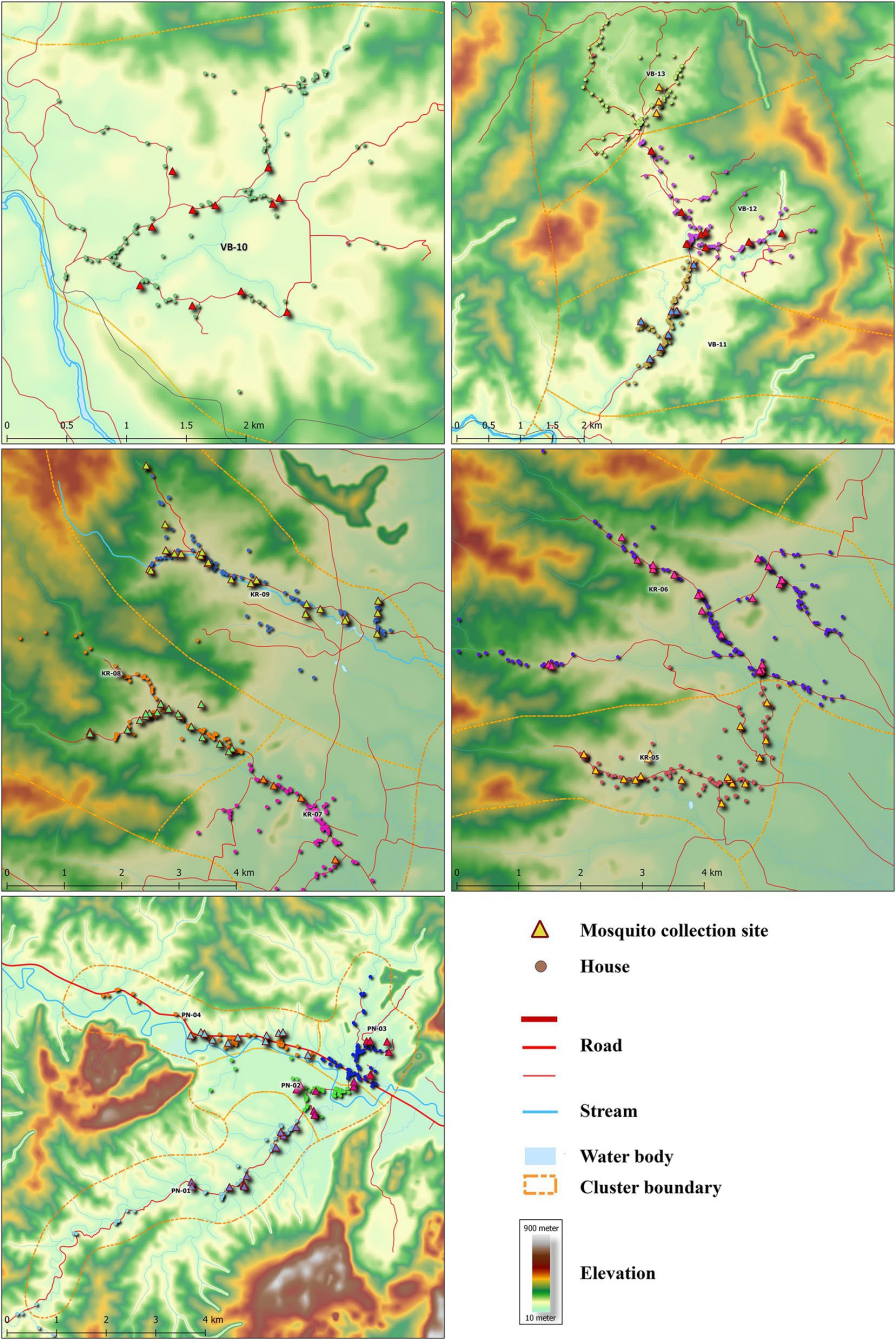
Enlarged (zoomed-in) terrain maps for each group of clusters surveyed in 2019. Houses (circles) and mosquito collection sites (triangles) are marked along with cluster boundaries (dashed lines). As much of the cluster as was safely accessible was surveyed for mosquito collections

A terrain map of Surat Thani province with the clusters evaluated from Phanom (PN), Khiri Rat Nikhom (KR) and Vibhavadi (VB) districts is shown in Fig. 1. Figure 2 is a zoomed-in version of Fig. 1, with the terrain maps depicting the house and mosquito collection locations for each of the 13 clusters evaluated in this study.

### Species composition and abundance

**Fig. 3:**
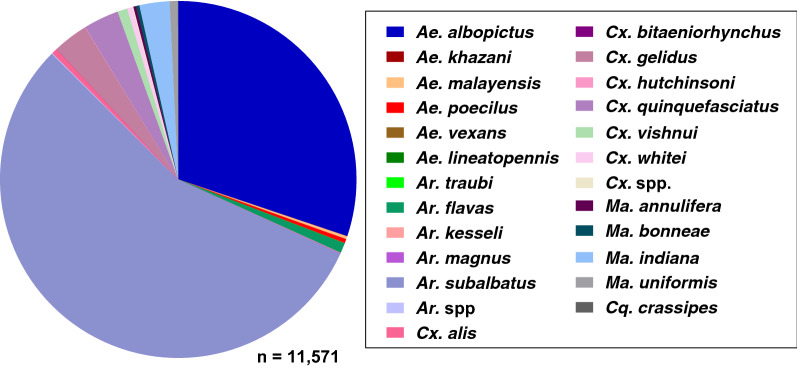
Proportion of the culicine species in the 11,571 mosquitoes captured from Surat Thani in 2019. *Ae*. *Aedes*, *Ar*. *Armigeres*, *Cq*. *Coquilletidia*, *Cx*. *Culex*, *Ma*. *Mansonia*

In 13 clusters from three districts, a total of 17,348 adult female mosquitoes were collected, representing six genera: *Armigeres* (37.78% of total female mosquitoes collected), *Anopheles* (33.32%), *Aedes * (20.58%), *Culex* (5.62%), *Mansonia* (2.69%) and *Coqullitettedia* (0.01%). A total of 23 culicine mosquito species were identified from 11,571 collected specimens, of which the most abundant were *Ar. subalbatus* (55.59%), *Ae. albopictus* (30.15%), *Cx. gelidus* (3.21%), *Cx. quinquefasciatus* (3.21%) and *Ma. indiana* (2.73%), with the remaining 18 culicine species each comprising < 1% of the total collection (Fig. 3).

**Fig. 4:**
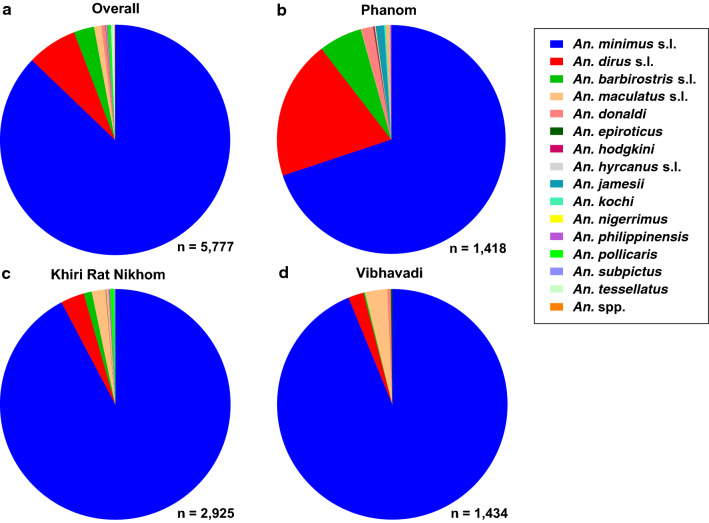
Proportion of *Anopheles* species identified morphologically overall (**a**) and by district (**b–d**)

Fifteen *Anopheles* species were morphologically identified from a total of 5777 specimens, of which ten belonged to the subgenus *Cellia* and five to the subgenus *Anopheles*. The predominant *Anopheles* captured at the study site were primary or suspected vectors and, listed in order of decreasing abundance, were: *Anopheles minimus* (s.l.) (87.17%; *n* = 5035), *An. dirus* (s.l.) (7.05%; *n* = 407), *An. barbirostris* (s.l.) (2.86%; *n* = 165) and *An. maculatus* (s.l.) (1.04%; *n* = 60). The remaining 11 species comprised 1.79% of the total *Anopheles* captured: *An. donaldi* (*n* = 32), *An. tessellatus* (*n* = 27), *An. pollicaris* (*n* = 23), *An. philippinensis* (*n* = 11), *An. subpictus* (*n* = 3), *An. epiroticus* (*n* = 2), *An. hodgkini* (*n* = 2), *An. nigerrimus* (*n* = 2), *An. hyrcanus* (s.l.) (*n* = 1), *An. jamesii* (*n* = 1) and *An. kochi* (*n* = 1). Only 0.09% (*n* = 5) of *Anopheles* specimens could not be morphologically identified to the species level (Fig. 4a).

Overall, *An. minimus* (s.l.) was the most abundant *Anopheles* species captured across all three districts, representing 69.89% of captured *Anopheles* species in Phanom, 92.27% in Khiri Rat Nikhom and 93.79% in Vibhavadi (Fig. 4a–d). The second and third most abundant *Anopheles* species by district were *An. dirus* (s.l.) and *An. barbirostris* (s.l.) in Phanom (19.68 and 6.14%, respectively) (Fig. 4b), *An. dirus* (s.l.) and *An. maculatus* (s.l.) in Khiri Rat Nikhom (3.32 and 1.91%, respectively) (Fig. 4c) and *An. barbirostris* (s.l.) and *An. dirus* (s.l.) in Vibhavadi (3.14 and 2.16%, respectively) (Fig. 4d). Of the total *Anopheles* captured (*n* = 5777), primary and secondary malaria vectors comprised 95.26% of the total *Anopheles* collected.

The Shannon–Wiener index and Simpson index were calculated per cluster for all species collected and for only the *Anopheles* species collected. For all species captured, no clear difference in mosquito diversity was found across the three districts, with the four clusters (PN-01, PN-04, KR-09, VB-10) of highest diversity occurring in each of the three districts. However, for *Anopheles* species, the four Phanom clusters had the highest diversity (Table 1).

**Table 1:** Total number of mosquitoes and *Anopheles* collected, and their respective Shannon–Wiener and Simpson indices, for each cluster

Number of mosquitoes and *Anopheles* collected and related indices	Cluster												
	PN-01	PN-02	PN-03	PN-04	KR-05	KR-06	KR-07	KR-08	KR-09	VB-10	VB-11	VB-12	VB-13
Total collected (*n*)	2026	822	1049	1051	1518	2518	689	2351	1576	946	614	1586	577
Total Shannon-Wiener Index	1.77	1.24	1.45	1.79	1.54	1.45	1.72	1.46	1.71	1.74	1.43	1.25	1.13
Total Simpson Index	4.45	1.91	2.88	4.35	3.32	3.23	3.90	3.50	4.08	3.98	3.18	2.91	2.63
*Anopheles* collected (*n*)	751	58	173	434	429	832	323	858	474	413	201	733	87
*Anopheles* Shannon-Wiener Index	2.07	3.25	1.52	1.49	1.20	1.20	1.24	1.11	1.13	1.20	1.32	1.04	1.29
*Anopheles* Simpson Index	0.97	1.36	0.69	0.67	0.45	0.42	0.43	0.25	0.32	0.39	0.53	0.11	0.48

Mosquitoes identified only to genera were not included in the totals or species diversity calculations

*KR* Khiri Rat Nikhom district,* PN* Phanom district, * VB* Vibhavadi district

The average number of *Anopheles* specimens collected per volunteer per night is shown in Table 2. Overall, nightly HLCs did not vary by district for all *Anopheles*, with an average of 11.46 *Anopheles* captured per collector per night. There were no differences in mean number of *An. minimus* (s.l.) captured per collector per night across the three districts. However, more *An. dirus* (s.l.) were captured per person per night from Phanom, and more *An. maculatus* (s.l.) were captured per person per night from Khiri Rat Nikhom, while fewer *An. barbirostris* (s.l.) were captured per person per night from Khiri Rat Nikhom (Table 2).

**Table 2:** Mean number of *Anopheles* specimens collected per volunteer per night by district

Study sites	*An. minimus* (s.l.)	*An. dirus* (s.l.)	*An. barbirostris* (s.l.)	*An. maculatus* (s.l.)	All *Anopheles*
PN	7.50 (3.50, 11.50)	**2.11 (1.02, 3.20)**	0.64 (0.34, 0.93)	0.01 (0.01, 0.02)	10.70 (5.95, 15.46)
KR	9.91 (7.88, 11.94)	0.35 (0.23, 0.47)	**0.12 (0.07, 0.17)**	**0.20 (0.10, 0.30)**	10.73 (8.64, 12.82)
VB	13.47 (7.13, 19.81)	0.31 (0.12, 0.50)	0.45 (0.18, 0.72)	0.03 (0.01, 0.06)	14.46 (8.14, 20.78)
All districts	9.99 (8.06, 11.92)	0.81 (0.49, 1.12)	0.32 (0.22, 0.42)	0.12 (0.06, 0.07)	11.46 (9.43, 13.50)

Values are presented as the mean with the 95% confidence interval (CI) in parentheses. Bolded values represent within-species CIs that do not overlap other the CIs of other districts

**Fig. 5:**
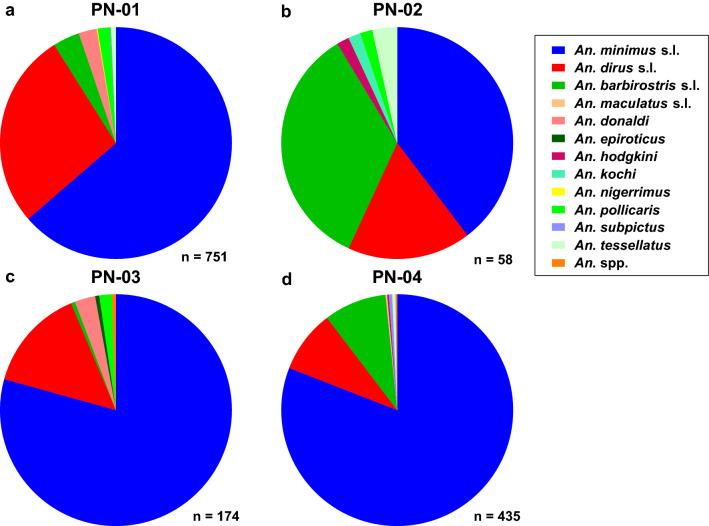
Morphological composition of *Anopheles* identified from the four clusters (**a**–**d**; *PN-01*–*PN-04*) in Phanom district

In Phanom, 12 *Anopheles* species were morphologically identified from four clusters. *Anopheles minimus* (s.l.) was the most abundant *Anopheles* species captured across all four clusters (Fig. 5a–d), and the second and third most abundant *Anopheles* species by cluster were *An. dirus* (s.l.) and *An. barbirostris* (s.l.) in PN-01 (Fig. 5a), *An. barbirostris* (s.l.) and *An. dirus* (s.l.) in PN-02 (Fig. 5b), *An. dirus* (s.l.) and *An.* *donaldi* in PN-03 (Fig. 5c) and *An. barbirostris* (s.l.) and *An. dirus* (s.l.) in PN-04 (Fig. 5d).

**Fig. 6:**
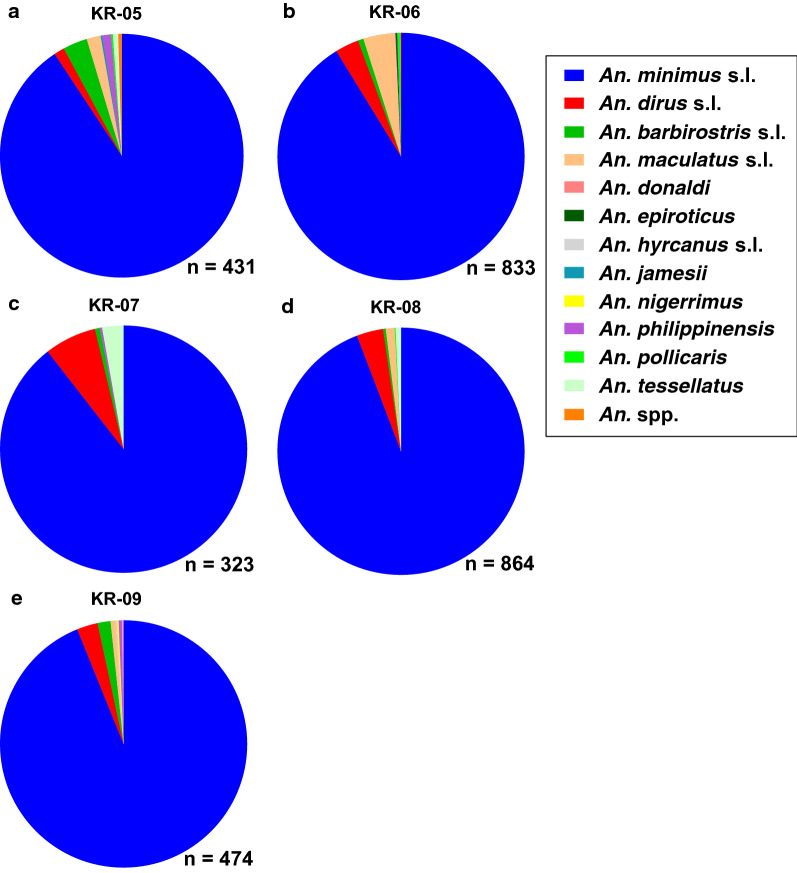
Morphological composition of *Anopheles* identified from the five clusters (**a**–**e**; *KR-05*–*KR-09*) in Khiri Rat Nikhom district

In Khiri Rat Nikhom, 12 *Anopheles* species were morphologically identified from five clusters. *Anopheles minimus* (s.l.) was the most abundant *Anopheles* species captured across all five KR clusters (Fig. 6a–e), and the second and third most abundant *Anopheles* species by cluster were *An. barbirostris* (s.l.) and *An. maculatus* (s.l.) in KR-05 (Fig. 6a), *An. maculatus* (s.l.) and *An. dirus* (s.l.) in KR-06 (Fig. 6b), *An. dirus* (s.l.) and *An. tesselatus* in KR-07 (Fig. 6c), *An. dirus* (s.l.) and *An. maculatus* (s.l.) in KR-08 (Fig. 6d) and *An. dirus* (s.l.) and *An. barbirostris* (s.l.) in KR-09 (Fig. 6e).

**Fig. 7:**
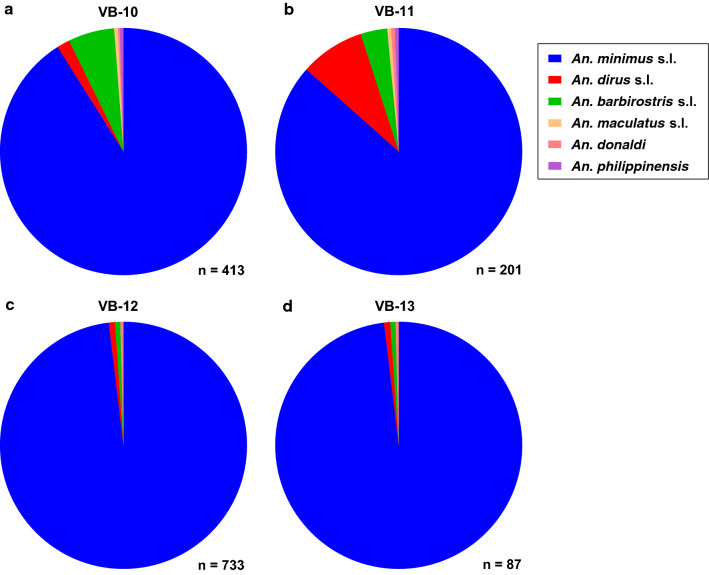
Morphological composition of *Anopheles* identified from four clusters (**a**–**d**;* VB-10*–*VB-13*) in Vibhavadi district

In Vibhavadi, only six *Anopheles* species were morphologically identified from four clusters. *Anopheles minimus* (s.l.) was the most abundant *Anopheles* species captured across all four VB clusters (Fig. 7a–d), and the second and third most abundant *Anopheles* species by cluster were *An. barbirostris* (s.l.) and *An. dirus* (s.l.) in VB-10 and VB-13 (Fig. 7a, d) and *An. dirus* (s.l.) and *An. barbirostris* (s.l.) in VB-11 and VB-12 (Fig. 7b, c).

A total of 879 *Anopheles* were identified molecularly to species level by AS-PCR or sequencing. Of the Funestus group, which contains the Minimus complex, 99.80% (*n* = 484) were *An. minimus* (s.s.) and 0.20% (*n* = 1) were *An. aconitus*. The Dirus complex was 100% (*n* = 347) *An. baimaii*. Of the Maculatus group, 93.62% (*n* = 44) were *An. maculatus* (s.s.) and 6.38% (*n* = 3) were *An. sawadwongporni*. One *An. epiroticus* and one *An. nigerrimus* specimen were identified by sequencing. The Barbirostris group members were identified to species level based on morphology. Of the Barbirostris group, 69.37% (*n* = 154) were *An. barbirostris*, 14.42% (*n* = 32) were *An. donaldi*, 10.36% (*n* = 23) were *An. pollicaris*, 4.95% (*n* = 11) were *An. campestris* and 0.90% (*n* = 2) were *An. hodgkini*.

### *Plasmodium* infection status

None of the 879 *Anopheles* mosquitoes tested were *Plasmodium* positive.

### *Anopheles* mosquito collection by time

**Fig. 8:**
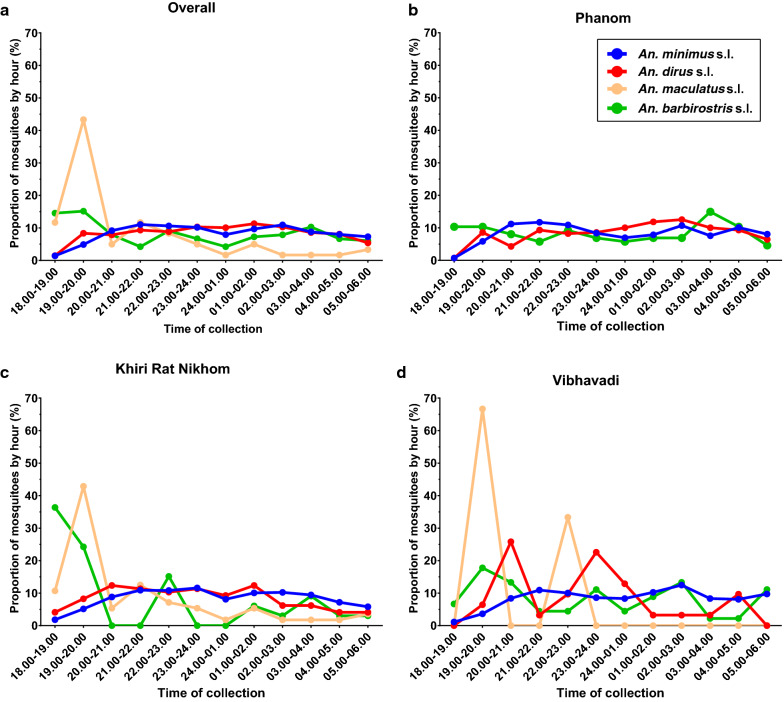
Proportion of *Anopheles* species collected by hour from all districts (**a**), Phanom (**b**), Khiri Rat Nikhom (**c**) and Vibhavadi (**d**)

Overall, there were significant differences in hour of collection by species (Kruskal–Wallis H-test: *χ*^2^ = 80.89, *df* = 3, *P* < 0.0001, *n* = 5666) with significantly more *An. barbirostris* (s.l.) and *An. maculatus* (s.l.) caught earlier in the night compared to *An. minimus* (s.l.) (Dunn’s multiple comparison test: *P* = 0.0001, *P* < 0.0001, respectively) and *An. dirus* (s.l.) (Dunn’s multiple comparison test: *P* = 0.0082, *P* < 0.001, respectively). Overall, almost 60% of *An. maculatus* (s.l.) and 30% of the *An. barbirsostris* (s.l.) were captured between 18:00 h and 20:00 h (Fig. 8a). In Phanom, there were no significant differences (Kruskal–Wallis H-test: *χ*^2^ = 6.892, *df* = 2, *P* = 0.0754, *n* = 1358) in time of *Anopheles* capture between the species (Fig. 8b), although too few *An. maculatus* (s.l.) (*n* = 1) were captured to be included in the analysis. In Khiri Rat Nikhom, there were significant differences in hour of collection by species (Kruskal–Wallis H-test: *χ*^2^ = 82.19, *df* = 3, *P* < 0.0001, *n* = 2884), with significantly more *An. barbirostris* (s.l.) and *An. maculatus* (s.l.) caught earlier in the night compared to *An. minimus* (s.l.) (Dunn’s multiple comparison test: *P* < 0.0001, *P* < 0.0001, respectively) and *An. dirus* (s.l.) (Dunn’s multiple comparison test: *P* = 0.0164, *P* < 0.001, respectively). In Vibhavadi, there were significant differences in hour of collection by species (Kruskal–Wallis H-test: *χ*^2^ = 21.61, *df* = 2, *P* < 0.0001, *n* = 1424), with significantly more *An. minimus* (s.l.) caught later in the night compared to *An. dirus* (s.l.) (Dunn’s multiple comparison test: *P* = 0.0108) and *An. barbirostris* (s.l.) (Dunn’s multiple comparison test: *P* = 0.0457). Too few *An. maculatus* (s.l.) (*n* = 3) were captured in Vibhavadi to be included in the analysis. Interestingly, there were significant differences in capture times across all three districts for *An. minimus* (s.l.) (Kruskal–Wallis H-test: *χ*^2^ = 16.66, *df* = 2, *P* = 0.0002, *n* = 5035), *An. dirus* s.l. (Kruskal–Wallis H-test: *χ*^2^ = 17.4, *df* = 2, *P* = 0.0002, *n* = 407) and *An. barbirostris* (s.l.) (Kruskal–Wallis H-test: *χ*^2^ = 12.61, *df* = 2, *P* = 0.0018, *n* = 165). *Anopheles minimus* (s.l.) were captured later in the night in Vibhavadi district compared to Phanom district (Dunn’s multiple comparison test: *P* = 0.0275) and Khiri Rat Nikhom district (Dunn’s multiple comparison test: *P* = 0.0002). *Anopheles dirus* (s.l.) were captured later in the night in Phanom compared to Khiri Rat Nikhom (Dunn’s multiple comparison test: *P* = 0.0023) and Vibhavadi (Dunn’s multiple comparison test: *P* = 0.0088). *Anopheles barbirostris* (s.l.) were captured earlier in the night in Khiri Rat Nikhom compared to Phanom (Dunn’s multiple comparison test: *P* = 0.0013) and Vibhavadi (Dunn’s multiple comparison test: *P* = 0.0273).

### *Anopheles* mosquito parity status

A total of 3509 *An. minimus* (s.l.) and 322 *An. baimaii* were dissected to determine parity. Across the three districts the proportion of parous *An. minimus* (s.l.) and *An. dirus* (s.l.) ranged from 51.71 to 71.64% and from 55.32 to 74.71%, respectively (Table 3).

**Table 3:** Number and proportion of parous and nulliparous *An. minimus* (s.l.) and *An. dirus* (s.l.) by district

Study sites	*An. minimus* (s.l.)				*An. dirus* (s.l.)			
	Parous (*n*)	Nulliparous (*n*)	Total (*n*)	Parity (%)	Parous (*n*)	Nulliparous (*n*)	Total (*n*)	Parity (%)
PN	287	268	555	51.71	130	105	235	55.32
KR	1437	569	2006	71.64	65	22	87	74.71
VB	651	297	948	68.67	18	12	30	60.00
Total	2375	1134	3509	67.68	213	139	352	60.51

**Fig. 9:**
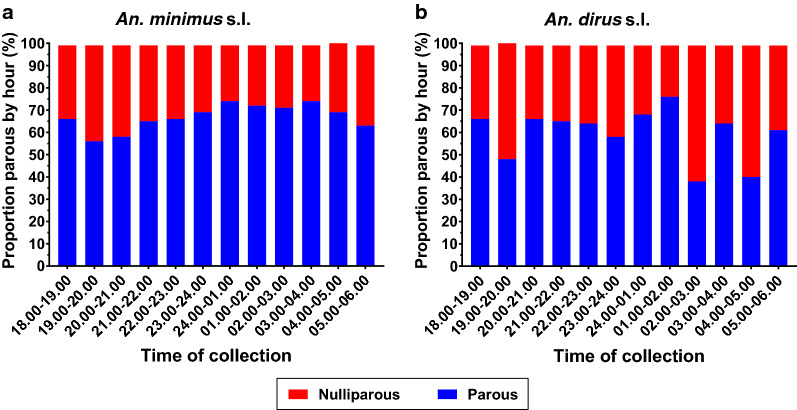
Proportion of *Anopheles minimus* (**a**) and *An. dirus* (**b**) that were parous or nulliparous collected per hour from all clusters with at least 100 dissected *An. minimus* and 20 dissected *An. dirus*

**Fig. 10:**
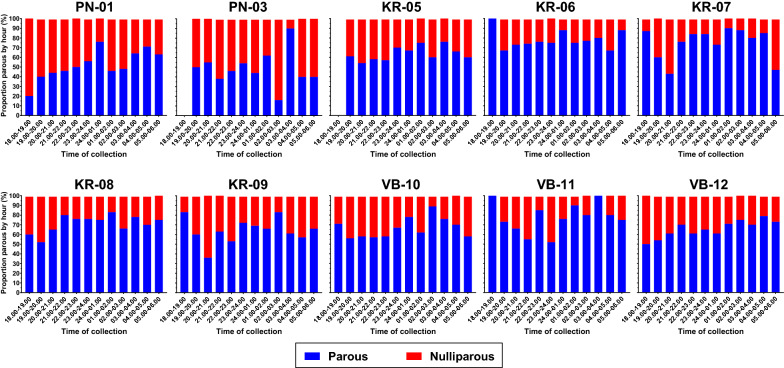
Proportion of *Anopeles minimus* (s.l.) that were parous or nulliparous collected per hour by cluster (*n* = 3400)

Overall, there was a significant increasing trend in the proportion of parous *An. minimus* (s.l.) captured by hour throughout the night [Wald Chi-square: *χ*^2^ = 17.31, *P* = 0.000, odds ratio (OR) 1.0535, 95% confidence interval (CI) 1.0279–1.0796, *n* = 3400] (Fig. 9a). Only clusters with > 100 dissected *An. minimus* (s.l.) were included in the analyses (i.e. PN-01, PN-03, KR-05, KR-06, KR-07, KR-08, KR-09, VB-10, VB-11, VB-12). While all clusters showed an increasing trend in the proportion of parous *An. minimus* (s.l.) throughout the night (Fig. 10), only PN-01 and VB-12 were significant (Table 4). For *An. dirus* (s.l.) there was no significant trend in the proportion of parous mosquitoes by hour throughout the night (Wald Chi-square: *χ*^2^ = 0.46, *P* = 0.497, OR 0.9702, 95% CI 0.8891–1.0587, *n* = 229) (Fig. 9b). Only clusters with > 20 dissected *An. dirus* (s.l.) (i.e. PN-01, PN-03, PN-04) were included in the analysis.

**Table 4:** Trend in parity by hour and cluster for *Anopheles minimus* (s.l.) (*n* = 3400)

Cluster^a^	*P*-value	OR (95% CI)	*n*
**PN-01**	**0.002**	**1.1083 (1.0393–1.1819)**	**392**
PN-03	0.680	1.0250 (0.9117–1.1523)	121
KR-05	0.251	1.0493 (0.9665–1.1393)	335
KR-06	0.438	1.0298 (0.9563–1.1089)	501
KR-07	0.884	1.0069 (0.9180–1.1044)	280
KR-08	0.102	1.0520 (0.9900–1.1180)	595
KR-09	0.250	1.0478 (0.9676–1.1347)	282
VB-10	0.530	1.0217 (0.9555–1.0925)	329
VB-11	0.133	1.1141 (0.9675–1.2829)	146
**VB-12**	**0.034**	**1.0824 (1.0059–1.1646)**	**419**

*n* number of mosquitoes dissected, *OR* odds ratio

^a^All clusters showed an increasing trend in the proportion of parous *An. minimus* (s.l.) throughout the night (Fig. 10), but the trend was only significant for PN-01 and VB-12 (bold)

### Power calculation

The purpose of this field study was to assess the potential of Surat Thani to serve as the location for future entomological evaluation of ivermectin MDAs. As *Plasmodium* infection rates in *Anopheles* in the GMS are extremely low, either survival or parity become the ideal metrics to measure. However, capturing indoor-resting, blood-fed *Anopheles* in the GMS is very difficult, rendering assessment of mosquito survival as done previously in Africa [12, 13] impossible. The power calculation to assess the impact of ivermectin MDA on mosquito parity was based on *An. minimus* (s.l.) as it is a primary malaria vector, the most abundant *Anopheles* species captured in Surat Thani (87.16%; 5035/5777) and the most ivermectin-sensitive malaria vector in the GMS evaluated to date [16, 17]. Parity rates (*P*_0_ = 67.68%; 2375/3509) from ten clusters wherein > 100 *An. minimus* (s.l.) were collected and dissected were used to calculate an intra-cluster correlation (ICC) value to be used for sample size calculations. Individual mosquito parity results were assessed with a random effects logistic regression model to obtain an ICC of 0. 0378. To demonstrate an effect, based on a two-sided * α* = 0.05, a parity rate at baseline and in control villages of *P*_0_ = 67.68%, a treatment effect size = 34% reduction, meaning treatment villages with a parity rate of *P*_1_ = 44.67% after MDA, and an ICC = 0.0378 requires at least four clusters per treatment arm and a minimum of 300 mosquitoes dissected per cluster to provide 82% power. A conservative treatment effect size of 34% was predicted based on a previously developed model for ivermectin MDA [41] parameterized with *An. minimus* (s.s.) ivermectin susceptibility data [17]. This treatment effect size is reasonable and supported by the following: (i) ivermectin MDAs in West Africa reduced the parity rate in *An. gambiae* by 30% [13]; (ii) *An. minimus* [7-day lethal concentration 50 (LC_50_) = 14.7 ng/ml) [17] is more susceptible to ivermectin than *An. gambiae* (7-day LC_50_ = 15.9 ng/ml) [42]; and (iii) we plan to administer 400 µg/kg ivermectin at our field site whereas 150–200 µg/kg was used in the West Africa MDA trials [13].

## Discussion

This study represents the most intensive and detailed analysis of *Anopheles* bionomics from Surat Thani province, Thailand. These surveys were conducted to determine which clusters in Surat Thani were most appropriate for evaluation of the entomological impacts of ivermectin MDA on *Anopheles*. Most (95.26%) of the *Anopheles* species collected from Phanom, Khiri Rat Nikhom and Vibhavadi districts were primary or secondary malaria vectors. In addition to detailed hourly collection data, this study presents data on the parity status throughout the night for the primary malaria vectors, *An. dirus* (s.l.) and *An. minimus* (s.l.). Finally, a power calculation was performed to determine the number of clusters that should be utilized to assess the impact of ivermectin MDA on *An. minimus* parity.

Surat Thani is unique in that it is a malarious province in Thailand that is not located along an international border. It has some of the highest rainforest coverage in the GMS and the highest rubber plantation coverage in Thailand, making it an ideal habitat for malaria vectors in the GMS. Indeed, of the 15 species captured in these surveys, 11 were either primary malaria vectors [*An*. *minimus* (s.l.), *An*. *dirus* (s.l.), *An*. *maculatus* (s.l.)], secondary malaria vectors [*An*. *epiroticus*] or suspected malaria vectors [*An*. *barbirostris* (s.l.), *An*. *hodgkini*, *An. hyrcanus* (s.l.), *An*. *nigerrimus*, *An*. *philippinensis*, *An*. *kochi*, *An*. *tessellatus*]. *An. minimus* (s.l.) accounted for 87.16% (5035/5777) and *An. dirus* (s.l.) 7.15% (407/5777) of the total *Anopheles* collected., respectively. Molecular identification confirmed that of all *An. minimus* (s.l.) collected, 99.80% were *An. minimus* (s.s.) (*n* = 484) and 0.2% were *An. aconitus* (*n* = 1), and of all *An. dirus* (s.l.) collected, 100% were *An. baimaii* (*n* = 348). Since the secondary and suspected vectors were captured in such low numbers, it is likely that the primary malaria vectors, *An. minimus* (s.s.) and *An. baimaii*, are largely responsible for *Plasmodium* transmission in the study area. However, as no specimens were *Plasmodium* positive (0/879), it cannot be confirmed that these vectors are solely responsible for transmission. A previous study in Ubon Ratchathani province, in northeastern Thailand, reported that suspected malaria vectors *An*. *barbirostris* (s.l.), *An*. *philippinensis* and *An*. *hyrcanus* (s.l.) are highly zoophagic, feeding mostly on cattle [43]. Since we only used the HLC method, this feeding preference may explain the low abundance of these species in the study area, making it difficult to rule them out as possible contributors to *Plasmodium* transmission in Surat Thani. One limitation of this study is the lack of assessment on the proximity of the mosquito collection sites to potential influential factors, such as the forest, larval habitat and livestock populations.

Primary malaria vectors in the GMS tend to be collected more frequently outdoors than indoors [9–11]. Since the aim of this study was to collect as many human host-seeking *Anopheles* as possible, all HLCs were performed outdoors. Results from a cross-sectional survey in Surat Thani in the same study area where HLCs were performed indicated that staying outdoors is a primary risk factor for asymptomatic *Plasmodium* carriage, suggesting that most of the transmission occurs outside the home [26]. This finding reinforces the potential usefulness of ivermectin MDA in the GMS as it can target the *Anopheles* malaria vector regardless of location or time.

Vibhavadi had the fewest number of *Anopheles* species collected, with only six species, and *Anopheles* species diversity was similar to that in Khiri Rat Nikhom, which were both lower than species diversity in Phanom (Table 1). This reduced *Anopheles* diversity in Vibhavadi could be due in part to seasonality and the limited sampling duration from July to October, while mosquitoes were collected from February to October in Khiri Rat Nikhom and Phanom. Another interesting point about mosquito collections in Vibhavadi was that *An. minimus* (s.l.) were captured later in the night compared to Phanom (*P* = 0.0275) and Khiri Rat Nikhom (*P* = 0.0002). This could be due to the production of durian in Vibhavadi as thermal fogging with malathion occurs at night-time for the control of several durian crop pests, in particular *Scirtothrips dorsalis* (Order Thysanoptera; Family Thripidae) and *Allocaridara malayensis* (Order Homoptera; Family Psyllidae), and this fogging was observed at some of the mosquito collection sites during HLCs in Vibhavadi clusters. Vibhavadi has the fourth highest coverage (1.33%) of durian plantation of the 19 districts in Surat Thani, with Khiri Rat Nikhom having the eighth highest coverage (0.33%) and Phanom the twelfth highest coverage (0.18%) [44].

The abundance of *An. dirus* (s.l.) varied among districts, with the greatest numbers captured in Phanom, likely due to geographical (Figs. 1, 2) and biological characteristics as the Phanom study site is surrounded by national parks comprised of primary old growth rain forest and steep hillsides. Furthermore, rubber plantations adjacent to the forest create a suitable habitat for *An*. *dirus* (s.l.) proliferation [7, 22]. *Anopheles dirus* (s.l.) were captured later in the night in Phanom compared to Khiri Rat Nikhom (*P* = 0.0023) and Vibhavadi (*P* = 0.0088), but this may have been an artifact due to the smaller number of *An. dirus* (s.l.) captured in Khiri Rat Nikhom and Vibhavadi districts.

These surveys recorded some members of the Barbirostris group in Surat Thani for the first time, including *An. hodgkini*, *An. donaldi* and *An. pollicaris*. However, it is possible that these species may have been identified as *An. barbirostris* (s.l.) previously as they are difficult to distinguish morphologically [27]. Future work in the study area should identify the members of the Barbirostris group by molecular methods as not all species in this group are malaria vectors [45]. *Anopleles sawadwongporni* was also recorded for the first time in Surat Thani, but this species may not have been identified in previous surveys due to lack of molecular species identification [27]. *Anopheles baimaii* is also a newly recorded species for Surat Thani, likely missed previously due to the lack of molecular species identification. It is somewhat surprising that more members of the Dirus complex were not identified because *An. dirus* (s.s.), *An. cracens*, *An. nemophilous* and *An. scanloni* have been observed in the adjacent provinces of Phang Nga, Krabi, Nakhon Si Thammarat and Ranong [7].

There were no *Plasmodium*-positive *Anopheles* (0/879) specimens detected. However, this is not very surprising as the likelihood of finding sporozoite-infected *Anopheles* in the GMS is very low, typically lower than 1:1000 [11]. This is why the population age structure (i.e. parity rate) was selected as the primary entomological outcome indicator to assess impact of ivermectin MDA on *Anopheles* populations in Surat Thani. This study found an overall *An. minimus* (s.l.) parity of 67.68% (2375/3509), which is comparable with the results of other surveys in Thailand [3, 4, 46, 47]. Similar to the results reported by Sithiprasana et al. [3], little fluctuation in *An. minimus* (s.l.) parity was observed from cluster to cluster, with an ICC value of 0.0378. For *An. minimus* (s.l.), there was a significant trend of increasing parity by hour of collection, suggesting that older *An. minimus* tend to feed later at night (Fig. 9a), but no trend was observed for *An. dirus* (s.l.) (Fig. 9b). A power calculation determined that an *An. minimus* (s.l.) parity reduction treatment effect size = 34%, with four clusters per treatment arm and a minimum of 300 mosquitoes dissected per cluster at an *α* = 0.05 will provide 82% power to detect a significant difference in the population age structure (i.e. parity). Due to reduced malaria cases and transmission in Surat Thani [26], evaluation of the *Anopheles* parity rate is expected to provide a valuable outcome to assess the impact of ivermectin MDA on malaria transmission.

## Conclusions

An abundance of *Anopheles* primary malaria vectors were captured in Surat Thani. This study illustrates that Surat Thani will be an ideal field site for evaluating the impacts of ivermectin MDA on local *Anopheles* population age structure.

## Data Availability

The datasets used and/or analyzed during the current study are available from the corresponding author on reasonable request.

## Abbreviations

AS-PCR: Allele-specific PCR
DMSO: Dimethyl sulfoxide
GMS: Greater Mekong Subregion
HLC: Human landing collection
HRPO: Human Research Protection Office
ICC: Intra-cluster correlation
IRS: Indoor residual spraying
KR: Khiri Rat Nikhom
LLINs: Long-lasting insecticide-treated nets
MDA: Mass drug administration
MEGA: Molecular evolutionary genetics analysis
MoPH: Ministry of Public Health
PN: Phanom
VB: Vibhavadi
WRAIR: Walter Reed Army Institute of Research
